# Dietary protein sources, gut microbiome, and puberty timing in children: findings from a cohort study

**DOI:** 10.1038/s41392-024-01890-5

**Published:** 2024-07-01

**Authors:** Yujie Xu, Jingyuan Xiong, Xiaoyu Wang, Fang He, Guo Cheng

**Affiliations:** 1 Laboratory of Molecular Translational Medicine, Center for Translational Medicine, Key Laboratory of Birth Defects and Related Diseases of Women and Children (Sichuan University), Ministry of Education, Maternal & Child Nutrition Center, West China Second University Hospital, Sichuan University, Chengdu, Sichuan P.R. China; 2 West China School of Public Health and West China Fourth Hospital and Healthy Food Evaluation Research Center, Sichuan University, Chengdu, P.R. China

**Keywords:** Preclinical research, Predictive markers

**Dear Editor**,

Developing countries are in a critical term of nutritional transition, marked by a sharp increase in the consumption of animal-source foods. In China, the intake of calories from animal-source foods in children tripled from 1990 to 2019, accompanied by a secular trend in early puberty. Early puberty is a topic of much research interest due to its associations with the risk of all-cause mortality, hormone-related cancers, and metabolic diseases.^1^ Recent evidence has shown that different dietary proteins can modulate the gut microbiome composition and thereby exert distinct impacts on host health.^2^ However, no previous study has addressed the link between dietary protein sources, gut microbiota features, and pubertal development, which has important implications regarding early puberty trends in Chinese children.

We conducted a prospective cohort study of children aged 6 to 8 years based on the ongoing Chinese Adolescent Cohort (CAC) study. A total of 1826 participants with stool and urine samples were included. Participants were categorized to tertiles based on their dietary protein intake, and the highest animal protein intake group has a mean animal protein intake of 59.4 g daily, which is exceeding the recommended intake. Multivariate analysis by linear models (MaAsLin) revealed that *Butyricicoccus*, *Enterococcus*, *Dorea*, and *Romboutsia* were positively associated with vegetable protein intake and that *unidentified_Saccharimonad* was enriched in the highest animal protein group (all false discovery rate-adjusted *P* < 0.05). These genera biomarkers enriched in the high vegetable protein intake group are known short chain fatty acids (SCFAs)-producing taxa. We hypothesize that SCFAs may be metabolic clues involved in the different function of vegetable protein and animal protein.

Next, we generated the total protein-microbial index (TPMI), animal protein-microbial index (APMI), and vegetable protein-microbial index (VPMI) to quantify dietary protein-associated gut microbiome features, based on these gut microbe biomarkers. Through Cox proportional hazard regression models adjusted for possible confounders, children in the highest APMI tertile were more likely to experience earlier menarche or voice break than those in the lowest APMI tertile, while children with a higher VPMI were more likely to reach menarche or voice break later than those with a lower VPMI (Fig. 1a). As the main vegetable protein source, dietary soy has been suggested to be related to later puberty timing in Chinese children.^3^ Thus, we conducted a subgroup analysis stratified by dietary soy intake categories (T3: >40 g/d vs. T1: <8 g/d), to determine whether the impact of vegetable protein-shaped microbial features on puberty timing depends on dietary soy. Interestingly, a significant interaction effect of dietary soy intake with the VPMI on puberty timing (*P* = 0.01) was found: a greater VPMI was associated with later puberty timing only among children with greater dietary soy intake. Although it remains unclear whether dietary soy exert its beneficial effects via gut microbiota, this interaction association may provide a more precise recommendation regarding vegetable protein intake from dietary soy.

**Fig. 1:**
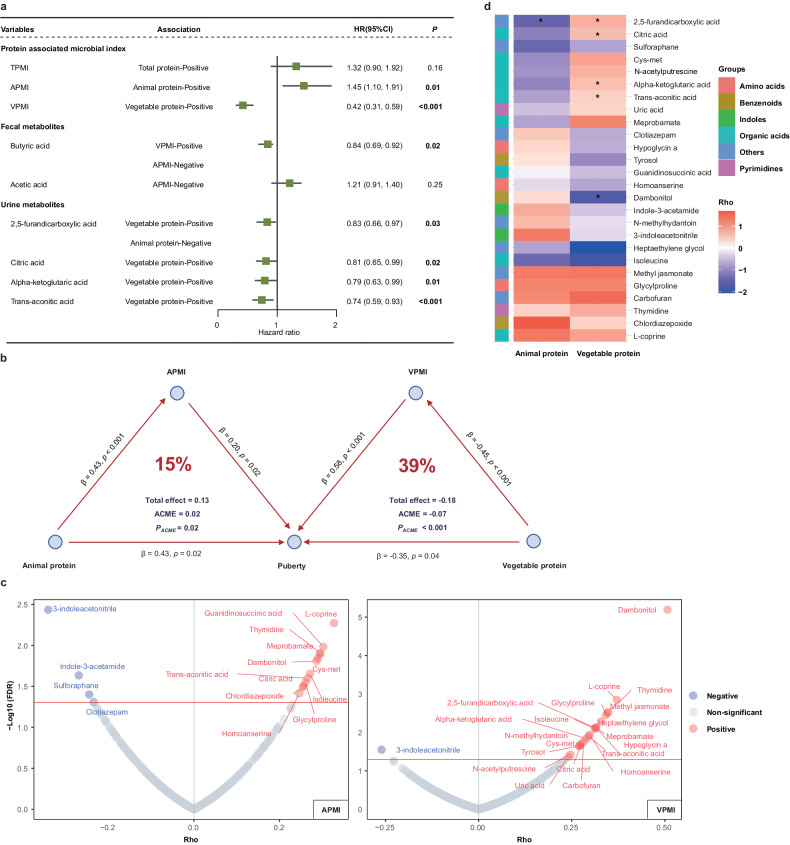
Associations among dietary protein intake, the gut microbiome, and puberty timing in Chinese children. **a** Associations of protein-associated microbial and metabolic features with puberty timing comparing the highest tertiles with the lowest tertiles by Cox regression analyses. TPMI, total protein-microbial index; APMI, animal protein-microbial index; VPMI, vegetable protein-microbial index; HR, hazard ratio; CI, confidence interval. **b** Mediation analyses of protein-microbial indices in the associations between different sources of protein and pubertal development. ACME, average causal mediation effect. **c** Urine metabolites linked to the animal protein-microbial index (left panel) or vegetable protein-microbial index (right panel) according to the partial correlation analysis. The x-axis shows the rho value, and the y-axis indicates the −log (base 10) of the FDR-corrected *P* value. APMI, animal protein-microbial index; VPMI, vegetable protein-microbial index. **d** The associations between urine metabolites and animal protein or vegetable protein consumption. An asterisk represents a significant association

Because the gut microbiome is shaped by habitual dietary intake, diet might exert significant impacts on pubertal maturation through modulation of the gut microbiota. Mediation analysis revealed that the APMI explained 15% of the total impact of animal protein intake on earlier puberty timing, while the VPMI contributed to 39% of the vegetable protein-puberty timing association (Fig. 1b). Some clues to this difference may lie in their unique digestibility. For instance, animal protein is highly digestible, resulting in less degradation via gut bacteria, while vegetable protein with low digestibility is more dependent on gut metabolism.

Notable differences in fecal and urine metabolites were also observed in the vegetable protein-shaped gut microbiome structure compared to the animal protein-shaped gut microbiome structure (Fig. 1c). Urine 2,5-furandicarboxylic acid, citric acid, alpha-ketoglutaric acid and trans-aconitic acid, and fecal butyric acid were positively associated with vegetable protein consumption, and fecal butyric acid and urine 2,5-furandicarboxylic acid were negatively associated with animal protein intake (Fig. 1d). Of them, butyric acid, 2,5-furandicarboxylic acid, citric acid, alpha-ketoglutaric acid, and trans-aconitic acid were associated with later puberty timing (Fig. 1a). The citric acid and alpha-ketoglutaric acid found in our study were enriched in the cycle pathway and glutamate metabolism pathway, which are reportedly associated with endocrine function and the activation of hormone-releasing hormone secretion,^4^ respectively. Intriguingly, puberty is characterized as a period of physiological insulin resistance, and adolescents tend to be more susceptible to metabolic changes. Butyric acid has a beneficial impact on glucose homeostasis,^5^ and thus may contribute to the regulation of pubertal development. Our findings collectively suggest that habitual intake of animal protein and vegetable protein has the potential to influence puberty timing by reshaping the gut microbiome in different directions.

The present study has several strengths. Based on the larger sample and representative age and economic status of the participants, reliable conclusions could be made. The prospective nature and relatively long-term follow-up, in conjunction with the ability to adjust for major potential confounders, are substantial strengths of this study. Notably, to the best of our knowledge, this is the first cohort study to examine the association of different protein types with overall gut microbial structure and their impact on puberty timing in children by applying a multi-omics method.

Some limitations also need to be noted. First, the observational design of our study could not establish causal relationships, and there might be residual confounders that we could not capture. Second, serum samples are preferred for use in host metabolite determination, whereas the inclusion of blood samples in large observational studies of children and adolescents is difficult. However, the urine samples would be a good alternative method for testing host metabolism and a superior approach for studying host-gut microbiota interactions. Finally, our study design and methodological strategy indicate that the detrimental effects of microbial profiles shaped by animal proteins on early pubertal development were present only in comparisons of intakes exceeding the recommended intake with normal intakes. In the framework of a balanced diet, it can be concluded that excessive consumption of animal protein can be harmful, but recommendations on its intake level cannot be made to balance potential advantages in terms of fertility and disadvantages in early puberty.

In conclusion, animal protein-associated and vegetable protein-associated gut microbial features and metabolites mediate the diverse direction of associations between corresponding protein intake and puberty timing. Together with the present findings, avoiding excessive animal protein intake and encouraging adequate vegetable protein intake from soy are important during nutritional transition.

### Supplementary information

Supplemental methods

## Data Availability

The de-identified dataset supporting the conclusions of this Article can be made available from the corresponding author upon reasonable request.

## References

[1] Golub MS (2008). Public health implications of altered puberty timing. Pediatrics.

[2] Li J (2022). Dietary protein sources, mediating biomarkers, and incidence of type 2 diabetes: findings from the women’s health initiative and the UK Biobank. Diabetes Care.

[3] Xiong J (2022). Prospective association of dietary soy and fibre intake with puberty timing: a cohort study among Chinese children. BMC Med..

[4] Roth CL, McCormack AL, Lomniczi A, Mungenast AE, Ojeda SR (2006). Quantitative proteomics identifies a change in glial glutamate metabolism at the time of female puberty. Mol. Cell Endocrinol..

[5] Machiels K (2014). A decrease of the butyrate-producing species Roseburia hominis and Faecalibacterium prausnitzii defines dysbiosis in patients with ulcerative colitis. Gut.

