# Supplementary material for: Dietary protein sources, gut microbiome, and puberty timing in children: findings from a cohort study
Source: Signal Transduct Target Ther. 2024 Jul 1;9:167. doi: 10.1038/s41392-024-01890-5 (PMC11214940; doi:10.1038/s41392-024-01890-5)
Supplement: Supplementary file 1 — Supplemental methods [file 41392_2024_1890_MOESM1_ESM.docx]

Supplementary Materials for

**Dietary protein sources, gut microbiome, and puberty timing in children: findings from a cohort study**

Yujie Xu^1^, Jingyuan Xiong^2^, Xiaoyu Wang^1^, Fang He^2^, Guo Cheng^1*^

*Corresponding author: [gcheng@scu.edu.cn](mailto:gcheng@scu.edu.cn)

**This PDF file includes:**

Supplementary materials and methods

**Supplementary Materials and Methods**

**Study participants**

Details of the CAC cohort were described elsewhere. Cooperative and voluntary children aged 6-8 in 23 selected schools were recruited yearly since 2013 and were followed up to their age of 15. Baseline information included socio-demographic issues, dietary intake and eating behaviors, physical activity and sedentary behaviors, anthropometry and pubertal development. Follow-up data were collected at regular intervals until 15: anthropometry and puberty assessments as well as urine and stool samples (since 2019) were conducted annually; dietary intake and physical activity data were collected biennially.

Between 2013 to 2018, 6967 children were included for baseline, and 5962 completed at least two follow-ups by 2021. A total of 1826 children provided their first morning urine samples or fecal samples in follow-ups at their B2/G2, respectively, after excluding children experiencing diarrhea, constipation or antibiotic treatment.

**Data collection**

A validated food frequency questionnaire which included 17 categories of 53 representative foods or food groups among local children was used to collect their dietary intakes over the past 12 months via face-to-face interviews. Dietary intakes were converted into energy and nutrient intakes using the NCCW software (V11.0, 2014), which reflects food composition in China. We calculated individual mean daily intakes of total dietary protein, animal protein (from red meat, poultry, fish and seafood, eggs and milk), and vegetable protein (from grains and soybeans).

According to Tanner stage standardized criteria, B2 and pubic hair (girls and boys) were assessed at each examination by investigators, and G2 was assessed by comparative palpation with a Prader orchidometer. Children were asked whether M or VB occurred; if so, respective months and years were recorded.

Height, weight, triceps skinfold thicknesses and subscapular angle sites were measured by investigators. Body mass index (BMI) sex- and age-independent standard deviation scores (SDS) were calculated based on Chinese reference curves. Data on frequency, duration and type of physical activity was collected by a validated physical activity questionnaire. Pregnancy and infancy information and domestic characteristics were provided by parents**.**

**Biological sample collection and profiling**

The first morning voided midstream urine samples and stool samples of children were collected by parents based on detailed instructions. All samples were stored in a refrigerator at -80℃.

Untargeted metabolomics of urine profiling was measured by liquid chromatography-tandem mass spectroscopy (LC-MS/MS), using high-resolution mass spectrometer Q Exactive HF (Thermo Fisher Scientific, USA) to collect both positive and negative ion data. The LC-MS/MS data processing was performed using the Compound Discoverer 3.1 (Thermo Fisher Scientific, USA) software, which is based on BMDB database, mzCloud and ChemSpider (HMDB, KEGG, LipidMaps) databases. A total of 3575 metabolites peaks were measured, and 1100 out of them were structurally identified.

Microbial DNA was extracted using TIANamp soil DNA kit (TIANGEN, Biotech, Beijing, China). The final DNA concentration and purity were monitored on 1% agarose gel electrophoresis. The 16S V3 and V4 regions of the microbial 16S rRNA gene were amplified with primers 341F: CCTAYGGGRBGCASCAG and 806R: GGACTACNNGGGTATCTAAT by PCR system. After the extraction from a 2% agarose, the PCR products were purified with Qiagen gel extraction kit (Qiagen, Düsseldorf, Germany). Purified amplicons were sequenced on an Illumina NovaSeq platform (Illumina, San Diego, USA) and 250 bp paired reads were generated. These sequence reads were then imported into the QIIME2 platform (V2.4) and errors were corrected by DADA2, which obtains amplicon sequence variants (ASVs). The taxonomy was classified using the Greengenes reference database at a 99% similarity.

Fecal volatile organic compounds were analyzed using a gas chromatography-MS method to quantify the microbiota-related short chain fatty acids (SCFAs). The SCFAs included acetic acid, propionic acid, butyric acid, isobutyric acid, valeric acid and isovaleric acid.

**Statistical analysis**

All the statistical analyses were performed using SAS version 9.4 and R version 4.0.2. Dietary protein and soy intakes were expressed as age-specific residuals from the regression of protein or soy intake on energy intake, and grouped into tertiles (T1- T3).

We calculated total protein-microbial index (TPMI), animal protein-microbial index (APMI) and vegetable protein-microbial index (VPMI) as new gut microbial features to summarize the gut microbes associated with protein variables. To investigate the prospective associations of baseline dietary protein intake with gut microbial features in B2/G2, we first used multivariate analysis by linear models (MaAsLin) to identify specific gut microbes at genus level associated with intake of total protein, animal protein and vegetable protein, adjusted for the same variables as above analysis. *P* values were adjusted by Benjamini Hochebrg method to control false discovery rate (FDR). Subsequently, we calculated a total protein-microbial index (TPMI), animal protein-microbial index (APMI) and vegetable protein-microbial index (VPMI) as new gut microbial feature to summarize the gut microbes associated with total protein intake or different protein type. The indices were calculated based on the relative abundance of these identified biomarkers and the formula was as below:

$I_{i}^{P}=\sum_{j=1}^{n} Aij$ $I_{i}^{N}=\sum_{j=1}^{m} Aij$ $X_{i}=\left( \frac{I_{i}^{P}}{n}-\frac{I_{i}^{N}}{m} \right)$*10000

$$TPMI/APMI/VPMI=\frac{X_{i}-\bar{X}}{\mathrm{SD}}$$

Aij: the relative abundance of every microbe (significantly related to dietary protein) identified by MaAsLin; $I_{i}^{P}$: a subset of the positively associated biomarkers in these gut microbes; $I_{i}^{N}$: a subset of the negatively associated biomarkers in these gut microbes; $\bar{X}$: the mean of $X_{i}$and $\mathrm{SD}$: the standard deviation of $X_{i}$. Finally, we applied a linear regression analysis to test whether these indexes were associated with each of the dietary protein intake variables after multivariable adjustment.

Linear regression models were conducted to determine the prospective association between habitual dietary protein intake and gut microbiota. Cox proportional hazard regression models were used to investigate the prospective relevance of dietary protein (and its types) intake at baseline with age at B2/G2 or M/VB, and the prospective association of protein-microbial indices at B2/G2 with age at M/VB. Multivariable models were adjusted for suspected cofounders selected in univariate analyses that altered the estimate by more than 10%, i.e., family income level, mother’s age at menarche, energy intake, soy intake (residuals), and BMI SDS at baseline. Hazard ratios (HRs) or β estimate and their 95% confidence intervals (CIs) were estimated by comparing the 2^nd^ and 3^rd^ tertiles to the 1^st^ tertiles in these models. Mediation analysis using the R package *mediation* were performed to investigate the causal role of gut microbiota in contributing to the association between dietary protein and pubertal development, and the sensitivity analysis were conducted to confirm the total effect, direct effect, and indirect effect (ACEM). Moreover, we conducted subgroup analysis stratified by dietary soy intake categories (T3: > 40 g/d vs T1: < 8 g/d) to examine the interaction of dietary soy for association between the protein-microbial indices and pubertal development.

The partial Spearman correlation analysis was conducted to identify metabolites in fecal and urine associated with TPMI, APMI and VPMI, and the association of the above-identified metabolites with corresponding dietary protein type intakes and puberty timing were examined using the same models and cofounders.
